# Nematicide Chalcones Act Synergistically on *Caenorhabditis elegans* and *Meloidogyne incognita* Without Disrupting Soil Microbial Diversity and with Limited Toxicity to Human Cells

**DOI:** 10.3390/molecules30173624

**Published:** 2025-09-05

**Authors:** Alejandro Calderón-Urrea, Shantanu Shinde, Sosse Kendoyan, Vukasin M. Jovanovic, Seungmi Ryu, Carlos A. Tristan

**Affiliations:** 1 Department of Biology, California State University, Fresno, CA 93740, USA; shantanujshinde@gmail.com (S.S.); soskendoyan@mail.fresnostate.edu (S.K.); 2 Department of Biology, Madera Community College, Madera, CA 93638, USA; 3 Stem Cell Translation Laboratory (SCTL), National Center for Advancing Translational Sciences (NCATS), National Institutes of Health (NIH), 9800 Medical Center Drive, Rockville, MD 20850, USA; vukasin.jovanovic@nih.gov (V.M.J.); seungmi.ryu@nih.gov (S.R.); carlos.tristan@nih.gov (C.A.T.)

**Keywords:** synergistic effects, chalcone combinations, control of PPN (Plant Parasitic Nematodes), pesticide safety, pesticides and alteration of soil microbiota

## Abstract

Plant Parasitic Nematodes (PPNs), such as *Meloidogyne incognita*, cause significant agricultural losses worldwide. Conventional nematicides like methyl bromide are being phased out due to environmental and health concerns, prompting the search for safer alternatives. In previous studies, chalcones **17**, **25**, and **30**, flavonoid compounds, were shown to effectively kill the model nematode *Caenorhabditis elegans* at concentrations of 10^−4^ M. However, the potential of these chalcones to act synergistically at lower concentrations has not been explored. In this study, the nematicidal efficacy of chalcones **17**, **25**, and **30** was evaluated individually and in combination at concentrations as low as 10^−6^ M. The results demonstrate a strong synergistic effect, with combinations achieving 90–100% mortality in *C. elegans* within 3 days. Additionally, the combination index method revealed significant toxic effects against *M. incognita* with chalcones **17** and **30** in binary and ternary combinations. To assess the effects of these chalcones on nontarget organisms, chalcones were also tested for antimicrobial activity against soil bacteria; analysis of soil microbiota using 16S rRNA sequencing indicated that chalcones did not significantly disrupt microbial populations. Furthermore, tests on human pluripotent stem cells (hPSCs) reveal no major effects on the viability of these cells at concentrations as high as the concentrations needed to kill nematodes. These findings highlight the potential of chalcones **17**, **25**, and **30** for effective nematode control without harming soil bacteria or human cells.

## 1. Introduction

Nematodes, commonly known as roundworms, represent one of the most diverse animal phyla, with an estimated 28,000 described species, of which over 16,000 are parasitic [1]. Among these, plant-parasitic nematodes (PPNs) are of particular significance, as they inflict devastating damage to global agriculture. Annual crop losses attributed to PPNs are estimated to reach $125 billion, underscoring their economic and ecological impact [2]. Among PPNs, *M. incognita* is a significant agricultural pest infecting a broad range of crop species and causing substantial economic losses [3]. This obligatory parasite infects the roots of a wide range of host plants, causing the formation of characteristic galls at feeding sites. These galls result from the transformation of plant cells into multinucleate giant cells, which serve as the nematode’s sole nutrient source [4]. The life cycle of *M. incognita* begins with second-stage juveniles (J2), which hatch from eggs under favorable conditions and invade host plant roots [5]. The development and proliferation of these nematodes severely compromise plant health, reducing yield and quality.

Control strategies for PPNs have historically relied on chemical nematicides, crop rotations, thermal treatments, and breeding resistant cultivars [6]. However, the widespread use of chemical nematicides has raised significant environmental and public health concerns. For instance, compounds such as 1,2-dibromo-3-chloropropane (DBCP) and ethylene dibromide were withdrawn from markets due to their deleterious effects on human health and the environment [7]. Similarly, aldicarb, a highly toxic nematicide, has been detected in groundwater, raising alarms about its safety [8]. Methyl bromide, once a widely used fumigant, was found to be a potent ozone-depleting substance, leading to its phase-out under international agreements [9].

Given these challenges, the search for sustainable and environmentally friendly alternatives to synthetic chemical nematicides has gained momentum. Natural products, such as chalcones, have emerged as promising candidates for nematode control. Chalcones, precursors in the flavonoid biosynthetic pathway, are naturally occurring compounds known for their diverse biological activities, including antibacterial, antitumor, antimalarial, antioxidant, and nematicidal effects [10]. Various studies have demonstrated the efficacy of chalcones against PPNs. For example, trans-1,3-diphenylpropenone (E-chalcone) has shown 100% lethality against *Globodera pallida* and *G. rostochiensis* at concentrations below 50 µM [11]. Similarly, chalcone derivatives have exhibited potent nematicidal activity against other nematode species, such as *Bursaphelenchus xylophilus* [12]. More recently, studies using *Caenorhabditis elegans* as a model organism have provided insights into the nematicidal properties of chalcones. Work from our laboratory has demonstrated that certain organic chalcones can induce 100% lethality in *C. elegans* at micromolar concentrations [13]. Moreover, the lipophilicity of chalcones appears to play a critical role in their nematicidal activity, influencing their ability to penetrate nematode cell membranes. *C. elegans* serves as an ideal model for studying nematicidal activity due to its well-characterized biology, rapid life cycle, and ease of maintenance in laboratory settings.

The concept of chemical synergy, wherein the combined effect of multiple compounds exceeds the sum of their individual effects, has garnered attention in nematicidal research [14]. Synergistic interactions can enhance the efficacy of nematicidal agents while minimizing the required dosages, thereby reducing environmental impact. However, genuine synergistic interactions are relatively rare and often concentration-dependent [15].

**Figure 1:**
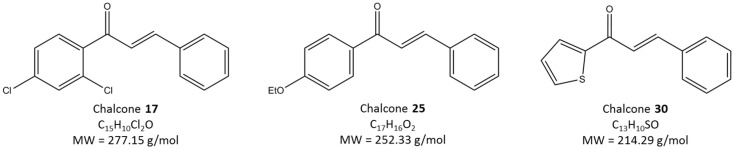
Molecular structure of Chalcones **17**, **25** and **30**.

Studies in Dr. Calderón-Urrea’s lab have indicated that chalcones **17**, **25**, and **30** [see Figure 1. Chalcone **17**: (2E)-1-(2,4-Dichlorophenyl)-3-phenyl-2-propen-1-one; Chalcone **25**: (2E)-1-(4-Ethoxyphenyl)-3-phenyl-2-propen-1-one; and Chalcone **30**: (2E)-3-Phenyl-1-(2-thienyl)-2-propen-1-one] exhibit nematicidal effects at concentrations of 10^−4^ M [13]. Preliminary findings indicate that these chalcones have effects at even lower concentrations (10^−5^ M), and when combined achieve near-complete lethality in *C. elegans* and *M. incognita*, suggesting potential synergistic interactions.

The combination index (CI) equation, based on the median-effect equation, provides a quantitative framework for evaluating such interactions [16]. This study aims to investigate the nature of interactions between chalcones **17**, **25**, and **30**, focusing on their effects on *C. elegans* and *M. incognita*. We hypothesize that the combined use of chalcones **17**, **25**, and **30** will exhibit a synergistic effect, resulting in enhanced lethality against both nematode species. By addressing these hypotheses, this study aims to provide insights into the potential of chalcone-based formulations as environmentally friendly nematicidal alternatives.

Soil is one of Earth’s most biologically diverse environments, hosting an array of microorganisms that play essential roles in nutrient cycling, organic matter decomposition, and maintaining soil quality [17]. The composition and diversity of soil microorganisms vary with location, vegetation, and resource availability. This variability underscores the importance of assessing the impact of chalcone treatments on specific microbial communities in soils affected by PPNs. Introducing chalcones into these systems could alter microbial diversity and abundance, potentially affecting soil quality and health [18]. Therefore, this study also aims to evaluate the effects of chalcones—**17**, **25**, and **30**—and their equimolar combinations on soil microbial communities. By evaluating the impact of chalcones on soil microorganisms, this study aims to determine whether these compounds can provide sustainable nematode control without compromising soil health. The findings will inform future risk assessments and guide the development of environmentally friendly pest management strategies. Finally, the chalcones were tested for cytotoxicity on two human pluripotent stem cells (hPSCs) revealing no major effects on the viability of these cells at concentrations as high as the concentrations needed to kill nematodes.

## 2. Results

### 2.1. Nematicidal Activity and Synergistic Interactions of Chalcones in C. elegans

**Figure 2:**
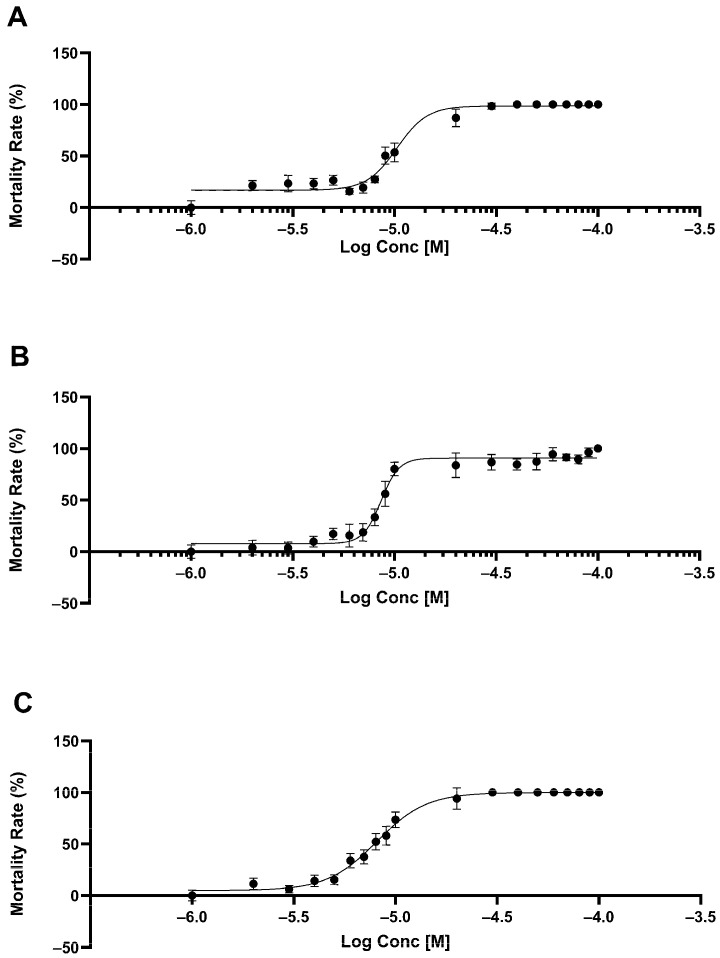
Percent mortality in *C. elegans* caused by Chalcone **17** (**A**), Chalcone **25** (**B**) and Chalcone **30** (**C**).

To evaluate the nematicidal effects of chalcones, compounds **17**, **25**, and **30** were assessed individually and in combinations for their ability to induce mortality in *Caenorhabditis elegans* after a 3-day exposure. Dose–response lethality tests identified approximate LD_30_ concentrations (dose treatment that will be lethal to approximately 30% of the population) for each chalcone by establishing concentration–mortality curves across 10^−4^ M to 10^−6^ M. Chalcones exhibited increasing mortality with higher concentrations, allowing LD_30_ identification in the 2–8 µM range. Chalcone **17** had LD_30_ values yielding 33–40% mortality, while chalcone **25** and chalcone **30** showed mortality in the 21–38% and 21–29% ranges, respectively (Figure 2).

Subsequently, pairwise (**17** + **25**, **25** + **30**, **17** + **30**) and ternary (**17** + **25** + **30**) combinations were tested at LD_30_ concentrations. If effects were additive, a ~60% mortality was expected. However, all combinations displayed synergistic interactions with mortality rates ≥ 93% and as high as 100%, significantly exceeding additive expectations (Table 1). Notably, the **17** + **25** combination at 8 µM yielded 97% mortality, confirming strong synergistic interactions. Mortality remained above 93% across all combinations even at 0.1 µM. The control (M9 buffer) mortality was < 20% (mean of all mixtures was 8% with a mean standard deviation of 0.023%), supporting the conclusion that the chalcone-induced effects were specific and significant.

### 2.2. Nematicidal Activity in Meloidogyne incognita

**Figure 3:**
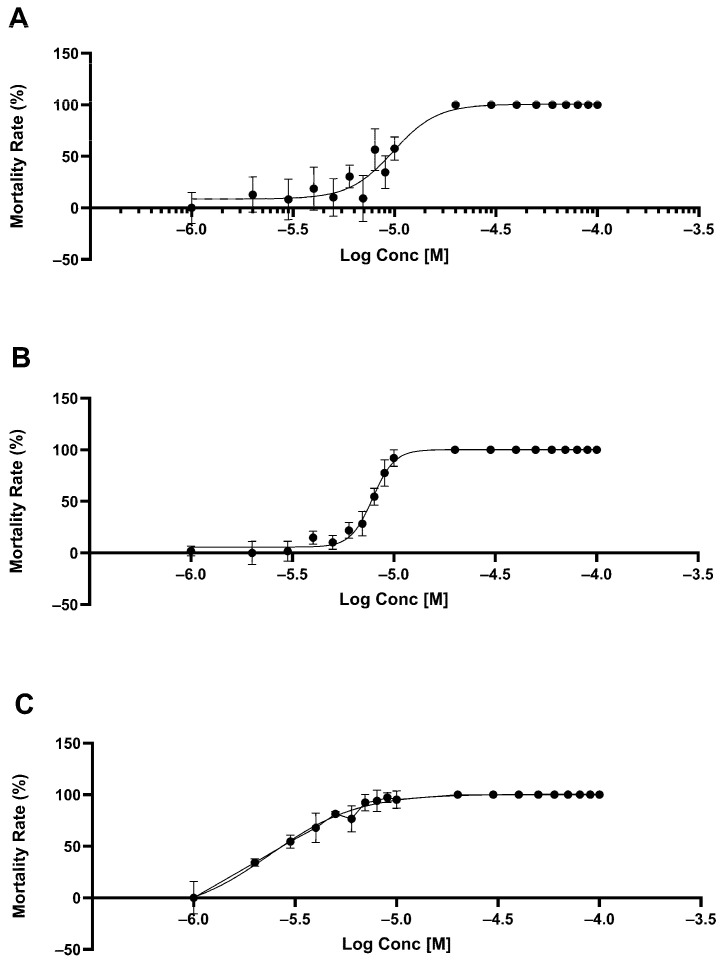
Percent mortality in *M. incognita* caused by Chalcone **17** (**A**), Chalcone **25** (**B**) and Chalcone **30** (**C**).

Chalcones **17**, **25**, and **30** were also evaluated for lethality in *M. incognita*, a major plant-parasitic nematode. At concentrations between 20 and 100 µM, all chalcones exhibited 100% lethality by day 5 post-treatment (Figure 3 and Table 2). Similarly to *C. elegans*, combinations of chalcones were tested. Synergistic lethality (>99%) was observed in combinations **17** + **25** + **30**, **25** + **30**, and **17** + **30** at concentrations of 4–10 µM. The **17** + **25** combination showed 100% lethality only at 10 µM and dropped below 90% at lower concentrations (Table 2).

**Figure 4:**
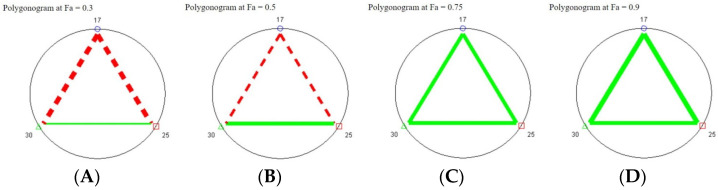
Polygonograms showing the interactions of three chalcones in binary combinations at four effect levels: (**A**) fa = 0.3, (**B**) fa = 0.5, (**C**) fa = 0.75, and (**D**) fa = 0.9. The interactions were calculated by CompuSyn 3.0.1 for *M. incognita*. Solid lines (green) indicate synergism, broken lines (red) indicate antagonism, and the thickness of the line represents the strength of synergism or antagonism. The figure shows synergistic interactions of (**25** + **30**) all over fa values, while combination (**17** + **25**) and (**17** + **30**) showing a dual synergistic/antagonistic interactions at low and high effect values, respectively.

To quantify the interaction effects, combination index (CI) values were calculated using the median-effect equation. Chalcone **17** had the lowest LD_50_ (0.52866 µM), indicating highest potency, while chalcone **25** had the highest LD_50_ (2.69874 µM). CI values confirmed synergism (CI < 1) in most combinations and concentrations, with a mild additive effect (CI ≈ 0.98) for 17 + 25 only at 1 µM. CI values across LD_30_ to LD_95_ showed consistent synergistic trends (Table 3 and Table 4). Polygonogram analysis supported these findings, with clear synergistic interactions observed across combinations and fractional effect levels (Figure 4).

### 2.3. Effects of Chalcones on Soil Bacterial Microbiota

To assess ecological safety, the effects of chalcones on soil microbiota were evaluated via 16S rRNA sequencing of samples treated with individual and combined chalcones. Alpha and beta diversity analyses were employed to characterize the microbial community structure across samples. Alpha diversity metrics provide insight into the diversity within individual samples. Two commonly used measures were applied in this study: (i) Observed OTUs, which quantify the total number of distinct operational taxonomic units (OTUs) detected, reflecting community richness without considering relative abundance (analogous to counting the number of different book titles in a library, irrespective of the number of copies), where higher values indicate a greater variety of microbial taxa; and (ii) the Shannon Diversity Index, which integrates both richness and evenness, such that higher values denote communities with a wide range of taxa distributed in a relatively balanced manner. To assess differences between microbial communities across samples, beta diversity metrics were calculated. Bray–Curtis dissimilarity was used to account for both species presence/absence and their relative abundances, with values ranging from 0 (identical communities) to 1 (completely distinct communities). Non-metric multidimensional scaling (NMDS) ordination was subsequently employed to visualize these differences, with samples plotted as points such that those in closer proximity represent more compositionally similar microbial communities, while those farther apart reflect greater dissimilarity.

**Figure 5:**
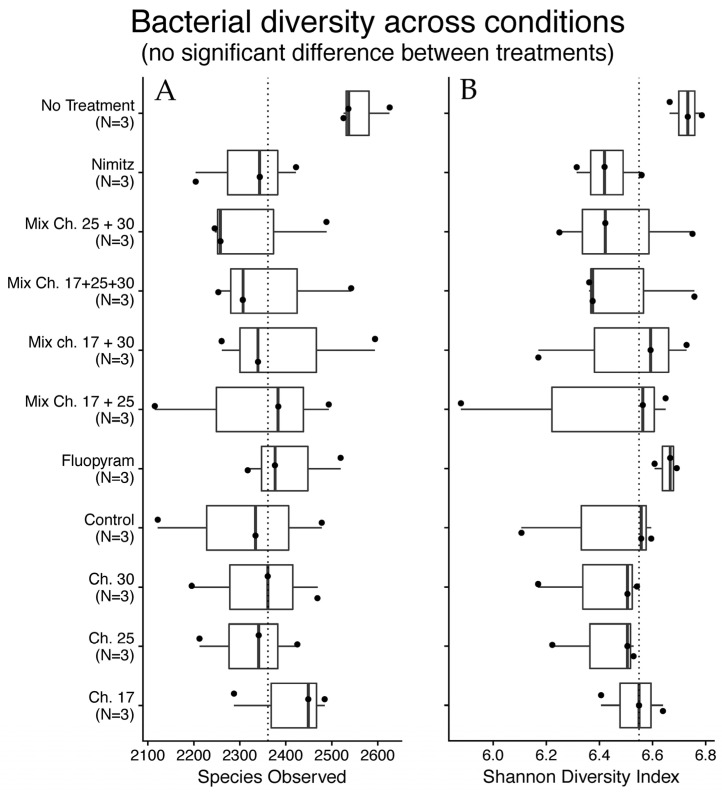
Bacterial diversity among sample treatments. The figure shows two boxplots representing the Observed OTU’s (**A**) and the Shannon index (**B**) of samples aggregated by sample treatment. The “Control” indicates a solvent control.

Alpha diversity metrics, including observed Operational Taxonomic Unit (OTUs) and Shannon diversity index, showed minimal disruption compared to untreated or Fluopyram-treated controls (Figure 5, Table 5 and Table 6). The untreated sample had the highest OTU count (2563 ± 55), with chalcone **17** (2407 ± 105) and Fluopyram (2404 ± 104) closely following. Among combinations, **17** + **30** and **17** + **25** + **30** preserved relatively high OTU diversity.

Shannon index values remained largely consistent across treatments, with the highest values in untreated soil (6.73 ± 0.06) and Fluopyram (6.66 ± 0.04), and slightly lower diversity observed in **17** + **25** (6.36 ± 0.42). Kruskal–Wallis pairwise comparisons revealed statistically significant differences (*p* < 0.05) in Shannon diversity for **17** + **25** + **30** vs. **17**, Fluopyram, and untreated samples, as well as between untreated samples and several other treatments (Table 7).

**Figure 6:**
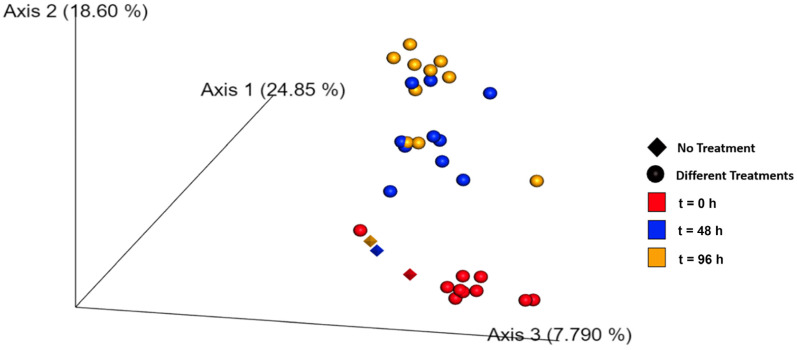
Graph representing the Beta Diversity results calculated using the Non-Metric Dimensional System (NMDS) ordination of Bray–Curtis Distances at t = 0, t = 48 and t = 96 h in the presence of chalcones **17**, **25**, **30** and their combinations.

Beta diversity assessed via NMDS ordination of Bray–Curtis dissimilarities (Figure 6) showed minimal shifts in microbial community composition. Temporal effects were minor and comparable across treatments. PERMANOVA analysis confirmed no significant differences between groups (*p* = 0.648), supporting the conclusion that chalcones do not induce major microbiome disruption.

### 2.4. Cytotoxicity of Chalcones in hPSCs

**Figure 7:**
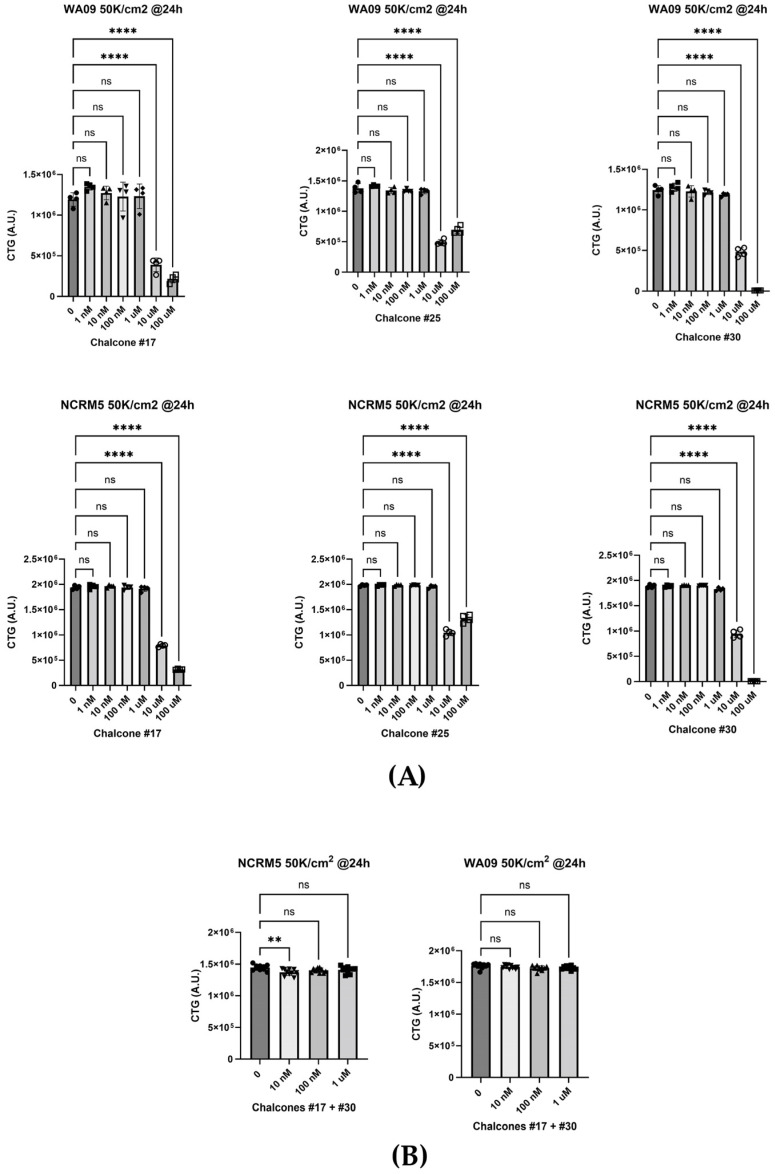
(**A**) effects of single chalcones on hPSCs and (**B**) effects of the chalcone **17** and chalcone **30** combination on hPSCs. n = 8. Error bar, mean ± SEM, one-way ANOVA with Dunnett’s multiple comparisons test, ** *p* < 0.01, **** *p* < 0.0001.

To investigate potential toxicity in human cells, two hPSC lines (WA09 and NCRM5) were treated with chalcones across a concentration range of 1 nM to 100 µM, and cell viability evaluated using the CellTiter-Glo (CTG) assay, which quantifies intracellular ATP as a surrogate for metabolically active, viable cells. All three chalcones were well tolerated up to 1 µM, with cytotoxic effects becoming evident only at 10 and 100 µM (Figure 7A). Furthermore, the **17** + **30** combination, effective in nematodes at micromolar concentrations (≤ 1 µM), was tested at 0, 10 nM, 100 nM, and 1 µM, and given that toxicity emerged in hPSCs above 1 µM, we restricted testing to 1 µM and below to ensure rigorous evaluation within its safe window in hPSCs. Cell viability remained unaffected, with only a minor reduction observed in NCRM5 at 10 nM (Figure 7B).

## 3. Discussion

This study provides a preliminary evaluation of the nematicidal potential, synergistic interactions, environmental safety, and cytotoxicity profile of three structurally related chalcones—**17**, **25**, and **30**—highlighting their promise as next-generation biopesticides. With growing concerns about food security and the ecological harm posed by traditional chemical nematicides, the need for effective and environmentally benign alternatives is increasingly critical [14]. Our findings provide preliminary evidence that these chalcones possess, nematode-specific lethality at low concentrations and exhibit enhanced efficacy when used in synergistic combinations, while exerting negligible effects on non-target organisms such as soil microbiota and hPSCs at concentration below 1 µM.

### 3.1. Synergistic Nematicidal Activity

It has been argued for a long time that the combination of insecticides with different modes of action is advantageous in integrated pest management (IPM) programs [20,21]. The synergistic lethality of chalcone combinations against both *Caenorhabditis elegans* and *Meloidogyne incognita* is a major highlight of this work. Individually, each chalcone was capable of inducing 100% mortality at 100 µM. However, combinations—particularly at lower micromolar levels (≤10 µM)—demonstrated markedly enhanced efficacy, frequently achieving mortality rates exceeding 90%. This synergism, confirmed by combination index (CI) analysis, suggests interactions that may involve distinct cellular targets or uptake pathways.

Effect-level dependency was evident in CI analysis: while some combinations (e.g., **25** + **30**) exhibited consistent synergy across all fractional effect (fa) levels, others (e.g., **17** + **25**, **17** + **30**, **17** + **25** + **30**) displayed a dual synergistic/antagonistic behavior, with antagonism at lower fa levels transitioning to synergy at fa > 0.6. These results underscore the importance of dosage context and support the rationale for using combination formulations to reduce active ingredient load while maintaining efficacy—an approach aligned with integrated pest management (IPM) principles [14].

### 3.2. Mechanistic Considerations

The precise mechanisms driving the synergistic toxicity remain to be elucidated, but may involve interactions affecting uptake, metabolism, or target binding. Previous studies have shown that chemical interactions can alter bioavailability, intracellular distribution, and elimination, thereby shaping toxicity profiles [15,22]. Others have shown that possible mechanisms of action involve the inhibition of P450 enzyme, which plays a critical role in the oxidation of several substances within the nematode [23]. The observed species-specific differences—robust synergy in *C. elegans* versus more variable responses in *M. incognita*—highlight the importance of test system selection and the need for broader cross-species analyses. Further studies employing molecular and genetic tools will be necessary to identify chalcone targets and to dissect the biochemical pathways implicated in nematode mortality.

### 3.3. Ecological Compatibility

Sustainable pest control strategies must avoid compromising soil health [24]. Our 16S rRNA-based microbiota analysis revealed that chalcone treatments—alone or in combination—did not significantly alter alpha (Shannon index) or beta (Bray–Curtis) diversity of soil bacterial communities. These findings suggest that the nematicidal effects of chalcones are highly selective, sparing the broader microbial ecosystem. While minor reductions in diversity were noted in certain treatments (e.g., **17** + **25** + **30**), they did not exceed thresholds of statistical or ecological concern (PERMANOVA *p* > 0.05).

Interestingly, a slight, non-significant enrichment of *Pseudomonas* species was observed. Given the dual role of *Pseudomonas* strains as both pathogens and plant growth promoters, further taxonomic resolution is warranted. Nevertheless, the limited ecological disruption seen here supports the compatibility of chalcones with long-term soil fertility and microbial function.

### 3.4. Cytotoxicity and Human Health Implications

Human toxicity is a critical consideration in biopesticide development [25]. Our assessment using two independent hPSC lines showed that chalcones, even at concentrations lethal to nematodes, exhibited minimal cytotoxicity, with only slight reductions in viability observed at the highest tested doses. This selective toxicity profile compares favorably with legacy nematicides such as DBCP and ethylene dibromide, which are associated with significant human health risks including carcinogenicity and reproductive toxicity.

The absence of observed toxicity from the chalcones, alone or in combination, on hPSCs at concentrations below 1 μM suggests a promising safety margin. However, a more comprehensive toxicity evaluation encompassing gene expression profiling, differentiation potential, and stress responses assays in hPSC-derived organoids and tissue models would further enhance our confidence on their safety profile and potentially provide insight toward the minimal but significant effects in viability observed in the NCRM5 hPSC line at 10 nM when treated with the **17** + **30** combination.

### 3.5. Limitations and Future Directions

While our findings support the efficacy and safety of chalcone-based nematicides, several limitations merit consideration. First, mechanistic studies are needed to elucidate the molecular targets of each chalcone and their interactions. Second, field trials in crop systems are required to validate efficacy under variable environmental conditions and to assess stability and degradation profiles. Additionally, long-term ecological studies—including impacts on non-target fauna and trophic interactions—will be essential to comprehensively establish environmental safety.

Finally, formulation development to enhance solubility, bioavailability, and delivery to target organisms will be key to transitioning chalcones from lab-scale evaluation to practical agricultural use.

## 4. Materials and Methods

### 4.1. C. elegans Culture and Chalcone Preparation

#### 4.1.1. *C. elegans* Strains and Maintenance

*Caenorhabditis elegans* strains were provided by the Caenorhabditis Genetics Center [CGC https://cgc.umn.edu/ (accessed on 4 September 2025)], which is funded by NIH Office of Research Infrastructure Programs (P40 OD010440). Wild-type *C. elegans* and the GFP-expressing strain PD4251 were cultured on nematode growth medium (NGM) agar seeded with *Escherichia coli* OP50. NGM plates were prepared by autoclaving a solution containing 17 g Difco^®^ agar, 12.5 g peptone, and 3 g NaCl per liter. After autoclaving, sterile additives included 1 mL cholesterol (5 mg/mL in ethanol), 1 mL 1 M CaCl_2_, 1 mL 1 M MgSO_4_, 1 mL 2 mg/mL uracil, and 25 mL 1 M potassium phosphate buffer (pH 6.0).

#### 4.1.2. Chalcone Stock Solutions and Dilution Series

Chalcones **17**, **25**, and **30** [synthesized as described [13] were each dissolved in dimethyl sulfoxide (DMSO) to obtain 0.1 M stock solutions. Serial dilutions were prepared by stepwise dilution in ethanol and M9 buffer, yielding final test concentrations ranging from 100 µM (10^−4^ M) to 1 µM (10^−6^ M). The final solvent concentrations were maintained below 0.1% DMSO and 5% ethanol to avoid toxicity to nematodes.

### 4.2. M. incognita Culture and Juvenile Isolation

#### Greenhouse Cultivation and Nematode Extraction

Tomato (*Solanum lycopersicum* cv. Moneymaker) plants were grown in sterilized sand and inoculated with *M. incognita*-infected roots. After 8–10 weeks, roots were harvested, chopped, and treated with 0.8% NaClO to release eggs, which were then isolated via sucrose flotation. Hatched second-stage juveniles (J2s) were collected using sterile hatching chambers with Kimwipe overlays and maintained at 25–27 °C until use.

### 4.3. Lethality Assays for Chalcones

#### 4.3.1. Single and Combination Treatments

96-well plate assays were performed using synchronized L3–L4 stage *C. elegans* or *M. incognita* J2s. For *C. elegans*, each well contained ~3 worms (picked nematodes from a healthy culture growing on OP50) and 100 µL of chalcone solution; in each experiment a minimum of 24 nematodes were treated. For *M. incognita*, ~50 J2s were exposed per well. Chalcones were tested singly and in combinations (**17** + **25**, **25** + **30**, **17** + **30**, **17** + **25** + **30**) at concentrations between 1 and 10 µM, chosen based on individual LD_30_ values. All assays (experiments) were performed in triplicate, and the mean and standard deviation of at least three trials were calculated for each treatment.

#### 4.3.2. Viability Assessment

*C. elegans*: Viability was determined via GFP fluorescence under a microscope; intact GFP localization indicated viability, while diffused GFP indicated mortality. *M. incognita*: Viability was assessed based on morphology and motility; dead worms appeared straight and immobile, while live worms showed active movement.

### 4.4. Combination Index and Synergy Analysis

The median-effect principle and combination index (CI) were used to quantify synergism, antagonism, or additive effects using CompuSyn 3.0.1 software (Combosyn Inc., Paramus, NJ, USA); see Figure S1 for the CompuSyn Report. Dose-effect curves were generated, and CI values were calculated at multiple effect levels (e.g., LD_30_, LD_50_, LD_90_). CI < 1 indicates synergy; CI = 1, additivity; and CI > 1, antagonism.

### 4.5. Effect of Chalcones on Soil Microbiota

#### 4.5.1. Experimental Design

To assess the ecological impact of chalcones, soil samples infected with *M. incognita* were treated with chalcones **17**, **25**, **30**, and their combinations at 10 µM in a greenhouse setting. Treatments included, untreated samples (no treatment), solvent controls (control), NIMITZ^®^ (ADAMA Ltd., Ashdod, Israel) and Fluopyram (Bayer AG, Leverkusen, Germany). Soil samples were collected at 0, 48, and 96 h post-treatment.

#### 4.5.2. PMA Treatment and DNA Extraction

To distinguish viable cells, soil samples were treated with propidium monoazide (PMA) prior to DNA extraction. DNA was extracted using the MoBio PowerSoil^®^ kit (QIAGEN, Germantown, MD, USA). Positive and negative controls included a known microbial community mixture and sterile soil.

#### 4.5.3. 16S rRNA Gene Sequencing and Microbiota Analysis

V4 regions of the 16S rRNA gene were amplified (using 515F-GTGCCAGCMGCCGCGGTAA and 806R-GGACTACHVGGGTWTCTAAT primers) and sequenced on the Illumina MiSeq platform (San Diego, CA, USA). QIIME2 version 2018.11 [26] was used to analyze alpha diversity (Shannon index, observed OTUs) and beta diversity (Bray–Curtis distances). Statistical significance was assessed using PERMANOVA.

### 4.6. Effect of Chalcones on hPSC

#### hPSC Cell Culture

hPSC lines WA09 and NCRM5 were cultured under feeder-free conditions in Essential 8 medium (A1517001, ThermoFisher, Waltham, MA, USA), on recombinant vitronectin (ThermoFisher, A14700) coated multi-well plates. When cells reached 70–80% confluency, cells were passaged using 0.5 mM EDTA (15575020ThermoFisher, Waltham, MA, USA) in phosphate-buffered saline at plating densities ranging from 25,000–50,000 cells per cm^2^ in E8 media supplemented with CEPT as previously described [27]. Twenty-four hours later, plating media was replaced with E8. The next day cells received a second media change and were treated with chalcones at the concentrations noted above. Twenty-four hours after treatment with chalcones, cell viability was measured using the CellTiter-Glo luminescent cell viability assay (G7570; Promega, Madison, WI, USA), according to the manufacturer’s recommendations.

## 5. Conclusions

Chalcones **17**, **25**, and **30** exhibit synergistic nematicidal activity at concentrations of approximately 12 µM (for example for the **17** + **30** combination), which can be considered as moderately potent. However, this is with minimal adverse effects on soil microbiota and human cells. These properties position them as promising candidates for sustainable, selective, and environmentally responsible biocontrol agents when used in combination; combinatorial use might also prevent the development of resistance against the chalcones. Their use could represent a significant advance in nematode management, reducing dependence on synthetic nematicides and contributing to safer agricultural practices.

## 6. Patents

US Pat No. 10,925,286 B2. Synergistic chalcone containing composition of a nematicide. February 23rd, 2021. https://patents.google.com/patent/US10925286B2/en?oq=10925286 (accessed on 4 September 2025).

US Pat No 11,369,111 B2. Synergistic composition of nematicide comprising chalcones. June 28th, 2022. https://patents.google.com/patent/US11369111B2/en?oq=US+Pat+No+11%2c369%2c111+B2 (accessed on 4 September 2025).

## Figures and Tables

**Table 1:** Percent mortality of individual, binary and ternary combinations of chalcones in *C. elegans*.

Percent Mortality of Individual and Chalcone Combinations							
µM	17	25	30	17 + 25	17 + 30	25 + 30	17 + 25 + 30
M9	7	7	10	7%	8%	10%	7%
10	62	81	78	100%	100%	100%	100%
8	40	38	60	97%	99%	96%	97%
6	31	21	44	99%	99%	99%	99%
4	37	23	28	99%	97%	93%	99%
2	35	10	25	93%	94%	94%	97%
1	-	-	-	94%	97%	92%	97%
0.1	-		-	93%	96%	89%	92%

**Table 2:** Percentage mortality at day 5 in binary and ternary combinations of chalcones for *M. incognita*.

Mean Mortality at Day 5								
µM	Solvent Control	17	25	30	17 + 25 + 30	17 + 25	25 + 30	17 + 30
Control	30.98							
10		83.61	95	97.61	100	100	100	100
8		83.22	71.35	97.05	100	88.65	100	100
6		73.09	50.68	88.33	100	86.07	99.01	100
4		68.58	46.32	84.11	99.11	79.27	90.32	96.10
2		66.36	36.90	67.35	88.90	65.55	76.93	85.01
1		61.37	38.12	50.36	65.91	52.90	55.85	66.06
0.1		-	-	-	59.42	30.88	48.08	51.72

**Table 3:** The dose-effect curve parameters (Dm, m and r) of the individual chalcone and their binary and ternary combinations, as well as the mean combination index (CI) values of the combinations.

	Dose-Effect Parameters			CI Values (Simulation)				
Chalcone Mixtures	Dm	m	r	LD30	LD50	LD75	LD90	LD95
**17**	0.52866	0.49929	0.91179					
**25**	2.69874	1.13849	0.75109					
**30**	1.17503	1.55255	0.96473					
**17** + **25**	1.25970	1.51818	0.84971	4.00226	1.42478	0.45544	0.20618	0.14499
**25** + **30**	1.10784	2.57209	0.89924	0.89603	0.67666	0.47598	0.33901	0.27084
**17** + **30**	0.96596	3.10792	0.95207	4.33634	1.32462	0.43256	0.22516	0.16558
**17** + **25** + **30**	0.85329	3.04931	0.97873	2.70836	0.88547	0.31404	0.16586	0.12014

Note: The parameters m, Dm (LD_50_) and r are the antilog of x-intercept, the slope and the linear correlation coefficient of the median–effect plot, which signifies the shape of the dose–effect curve, the effectiveness (LD_50_), and conformity of the data to the mass-action law, respectively (Chou and Talalay, 1984 [19]; Chou, 2006 [20]). LD_50_ and m are used for calculating the CI values; CI < 1, CI = 1, and CI > 1 indicate synergism, additive effect, and antagonism, respectively. LD_30_, LD_50_, LD_75_, LD_90_ and LD_95_ are the doses required to reach a response mortality of 30%, 50%, 75%, and 90%, respectively.

**Table 4:** CI values and their significance at actual data points.

	CI Value				Significance of CI Values
Conc. µM	17 + 25 + 30	17 + 25	25 + 30	17 + 30	
10	0.03604	0.03368	0.25329	0.04977	strong synergy (CI < 0.1)
8	0.02884	0.39745	0.04325	0.03982	synergy (0.1 > CI < 0.9)
6	0.02163	0.37529	0.15197	0.02986	additive (0.9 > CI < 1.1)
4	0.06780	0.49777	0.52095	0.22629	antagonism (1.1 > CI < 10)
2	0.20610	0.76261	0.53968	0.33706	strong antagonism (CI > 10)
1	0.42104	0.97838	0.52926	0.52809	

**Table 5:** Representing the average and standard deviation of Alpha diversity measured by the OTUs observed at t = 0, t = 48 and t = 96 h in the presence of chalcones **17**, **25**, **30** and their combinations. The “Control” indicates a solvent control. Abbreviations: AVG: average; STD: standard deviation.

	Observed OTUs				
Treatments	t = 0	t = 48	t = 96	AVG	STD
Control	2478	2334	2121	2311	179
Fluopyram	2519	2377	2317	2404	104
Mix (**17** + **25**)	2493	2383	2115	2331	195
Mix (**17** + **25** + **30**)	2542	2254	2307	2368	154
Mix (**17** + **30**)	2594	2339	2261	2398	174
Mix (**25** + **30**)	2489	2258	2246	2331	137
Nimitz	2422	2343	2204	2323	110
No Treatment	2537	2625	2526	2563	55
Ch. **17**	2485	2449	2287	2407	105
Ch. **25**	2424	2212	2341	2326	107
Ch. **30**	2469	2361	2196	2342	138

**Table 6:** Representing the average and standard deviation of Alpha diversity measured using the Shannon Diversity Index observed at t = 0, t = 48 and t = 96 h in the presence of chalcones **17**, **25**, **30** and their combinations. Abbreviations: AVG: average; STD: standard deviation.

	Shannon Diversity Index				
Treatments	t = 0	t = 48	t = 96	AVG	STD
Control	6.60	6.56	6.11	6.42	0.27
Fluopyram	6.69	6.67	6.61	6.66	0.04
Mix (**17** + **25**)	6.65	6.56	5.88	6.36	0.42
Mix (**17** + **25** + **30**)	6.76	6.37	6.36	6.50	0.22
Mix (**17** + **30**)	6.73	6.59	6.17	6.50	0.29
Mix (**25** + **30**)	6.75	6.42	6.25	6.47	0.25
Nimitz	6.31	6.56	6.42	6.43	0.12
No Treatment	6.66	6.79	6.73	6.73	0.06
Ch. **17**	6.64	6.55	6.41	6.53	0.12
Ch. **25**	6.53	6.22	6.51	6.42	0.17
Ch. **30**	6.54	6.51	6.17	6.41	0.21

**Table 7:** Kruskal–Wallis Pairwise comparison between treatments (Shannon diversity values) indicating that the differences observed were not statistically significant.

Group 1	Group 2	*p*-Value		Group 1	Group 2	*p*-Value
Ch **17**	Ch **25**	0.5127		Ch (**17** + **25**)	Ch (**17** + **25** + **30**)	0.5127
Ch **17**	Ch **30**	0.2752		Ch (**17** + **25**)	Ch (**17** + **30**)	0.8273
Ch **17**	Ch (**17** + **25**)	0.8273		Ch (**17** + **25**)	Ch (**25** + **30**)	0.8273
Ch **17**	Ch (**17** + **25** + **30**)	0.0495		Ch (**17** + **25**)	Control	0.8273
Ch **17**	Ch (**17** + **30**)	0.2752		Ch (**17** + **25**)	Fluopyram	0.2752
Ch **17**	Ch (**25** + **30**)	0.5127		Ch (**17** + **25**)	Nimitz	0.8273
Ch **17**	Control	0.8273		Ch (**17** + **25**)	No Treatment	0.0495
Ch **17**	Fluopyram	0.2752		Ch (**17** + **25** + **30**)	Ch (**17** + **30**)	0.5127
Ch **17**	Nimitz	0.2752		Ch (**17** + **25** + **30**)	Ch (**25** + **30**)	0.5127
Ch **17**	No Treatment	0.1266		Ch (**17** + **25** + **30**)	Control	0.0495
Ch **25**	Ch **30**	0.8273		Ch (**17** + **25** + **30**)	Fluopyram	0.0495
Ch **25**	Ch (**17** + **25**)	0.8273		Ch (**17** + **25** + **30**)	Nimitz	0.5127
Ch **25**	Ch (**17** + **25**+**30**)	0.5127		Ch (**17** + **25** + **30**)	No Treatment	0.0495
Ch **25**	Ch (**17** + **30**)	0.5127		Ch (**17** + **30**)	Ch (**25** + **30**)	0.8273
Ch **25**	Ch (**25** + **30**)	0.5127		Ch (**17** + **30**)	Control	0.2752
Ch **25**	Control	0.2752		Ch (**17** + **30**)	Fluopyram	0.1266
Ch **25**	Fluopyram	0.1266		Ch (**17** + **30**)	Nimitz	0.8273
Ch **25**	Nimitz	0.5127		Ch (**17** + **30**)	No Treatment	0.0495
Ch **25**	No Treatment	0.0495		Ch (**25** + **30**)	Control	0.5127
Ch **30**	Ch (**17** + **25**)	0.8273		Ch (**25** + **30**)	Fluopyram	0.5127
Ch **30**	Ch (**17** + **25** + **30**)	0.5127		Ch (**25** + **30**)	Nimitz	0.8273
Ch **30**	Ch (**17** + **30**)	0.5127		Ch (**25** + **30**)	No Treatment	0.5127
Ch **30**	Ch (**25** + **30**)	0.5127		Control	Fluopyram	0.5127
Ch **30**	Control	0.2752		Control	Nimitz	0.2752
Ch **30**	Fluopyram	0.1266		Control	No Treatment	0.2752
Ch **30**	Nimitz	0.8273		Fluopyram	Nimitz	0.1266
Ch **30**	No Treatment	0.0495		Fluopyram	No Treatment	0.2752
				Nimitz	No Treatment	0.0495

## Data Availability

The original metagenomic data presented in the study are openly available through NCBI under BioProject PRJNA1293377.

## Supplementary Materials

The following supporting information can be downloaded at: https://www.mdpi.com/article/10.3390/molecules30173624/s1, Figure S1: CompuSyn Report.
