# Supplementary material for: Nematicide Chalcones Act Synergistically on Caenorhabditis elegans and Meloidogyne incognita Without Disrupting Soil Microbial Diversity and with Limited Toxicity to Human Cells
Source: Molecules. 2025 Sep 5;30(17):3624. doi: 10.3390/molecules30173624 (PMC12430292; doi:10.3390/molecules30173624)
Supplement: Supplementary file 1 [file molecules-30-03624-s001.zip › molecules-3790713-supplementary.pdf]

CompuSyn Report

**Experiment Name:**	Four chalcone combinations
**Description**	Synergistic effect of chalcones on M. incognita

Drug: Chalcone (17) [uM]
Drug: Chalcone (25) [uM]
Drug: Chalcone (30) [uM]
Drug Combo: Chalcone (17+25) (17+25 [1:1])
Drug Combo: Chalcone (25+30) (25+30 [1:1])
Drug Combo: Chalcone (17+30) (17+30 [1:1])
Drug Combo: Chalcone 17+25+30 (172530) (17+25+30 [1:1:1])

Data for Drug: 17 [uM]

Dose	Effect
10.0	0.83
8.0	0.83
6.0	0.73
4.0	0.68
2.0	0.66
1.0	0.61

6 data points entered.

**X-int:**	-0.2768
**Y-int:**	0.13821 +/- 0.07782
**m:**	0.49929 +/- 0.11244
**Dm:**	0.52866
**r:**	0.91179

Data for Drug: 25 [uM]

Dose	Effect
10.0	0.95
8.0	0.71
6.0	0.5
4.0	0.46
2.0	0.36
1.0	0.38

6 data points entered.

**X-int:**	0.43116
**Y-int:**	-0.4909 +/- 0.34631
**m:**	1.13849 +/- 0.50036
**Dm:**	2.69874
**r:**	0.75109

Data for Drug: 30 [uM]

Dose	Effect
10.0	0.97
8.0	0.97
6.0	0.88
4.0	0.84
2.0	0.67
1.0	0.5

6 data points entered.

X-int: 0.07005
Y-int: -0.1088 +/- 0.14661
m: 1.55255 +/- 0.21182
Dm: 1.17503
r: 0.96473

Data for Drug Combo: 17+25 (17+25 [1:1])

Dose A	Effect
5.0+	0.99
4.0+	0.88
3.0+	0.86
2.0+	0.79
1.0+	0.65
0.5+	0.52

6 data points entered.

X-int: 0.10027
Y-int: -0.1522 +/- 0.32600
m: 1.51818 +/- 0.47102
Dm: 1.25970
r: 0.84971

Data for Drug Combo: 25+30 (25+30 [1:1])

Dose A	Effect
5.0+	0.99
4.0+	0.999
3.0+	0.99
2.0+	0.9
1.0+	0.76
0.5+	0.55

6 data points entered.

X-int: 0.04448
Y-int: -0.1144 +/- 0.43300
m: 2.57209 +/- 0.62561
Dm: 1.10784
r: 0.89924

Data for Drug Combo: 17+30 (17+30 [1:1])

Dose A	Effect
5.0+	0.999
4.0+	0.999
3.0+	0.999
2.0+	0.96
1.0+	0.85
0.5+	0.66

6 data points entered.

**X-int:**	-0.0150
**Y-int:**	0.04674 +/- 0.34554
**m:**	3.10792 +/- 0.49925
**Dm:**	0.96596
**r:**	0.95207

Data for Drug Combo: 172530 (17+25+30 [1:1:1])

Dose A	Effect
3.33333+	0.999
2.66667+	0.999
2.0+	0.999
1.33333+	0.99
0.66667+	0.89
0.33333+	0.66

6 data points entered.

**X-int:**	-0.0689
**Y-int:**	0.21011 +/- 0.22118
**m:**	3.04931 +/- 0.31957
**Dm:**	0.85329
**r:**	0.97873

Dose-Effect Curve for Drugs

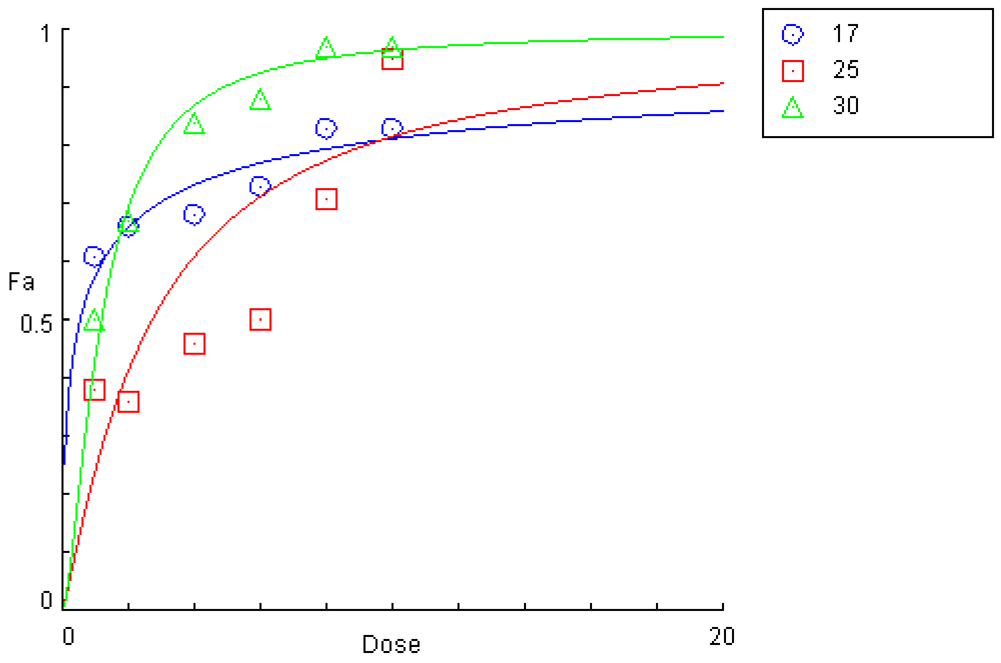

Dose-Effect Curve for Drug Combos

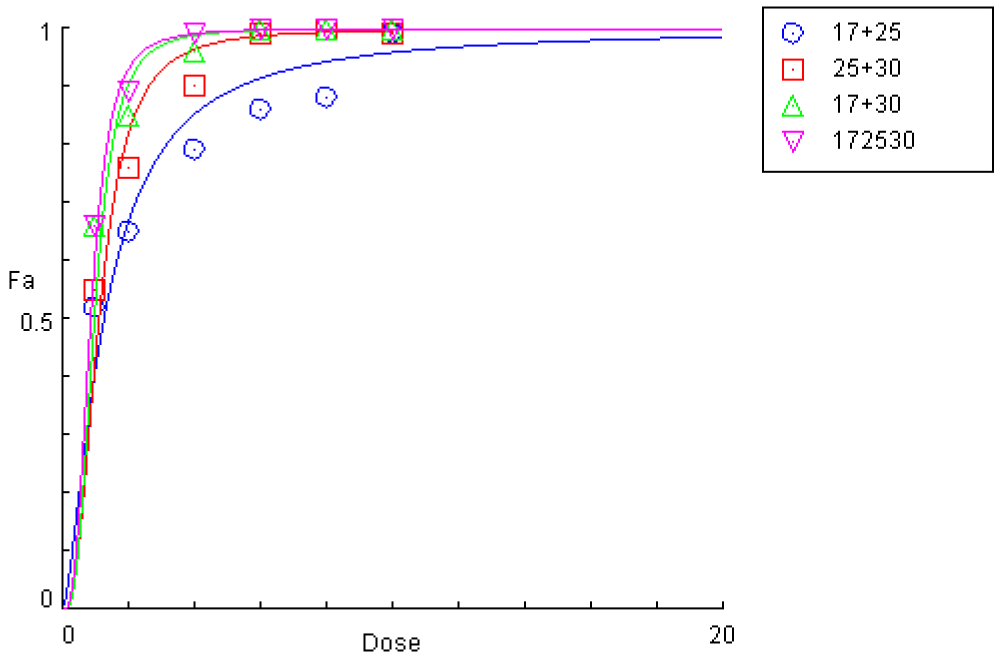

Median-Effect Plot for Drugs

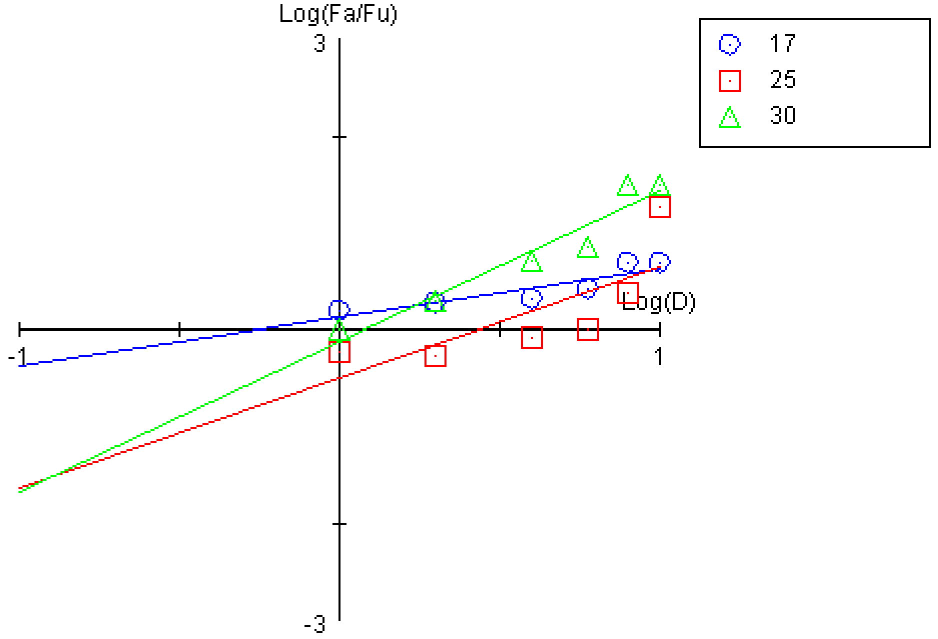

Median-Effect Plot for Drug Combos

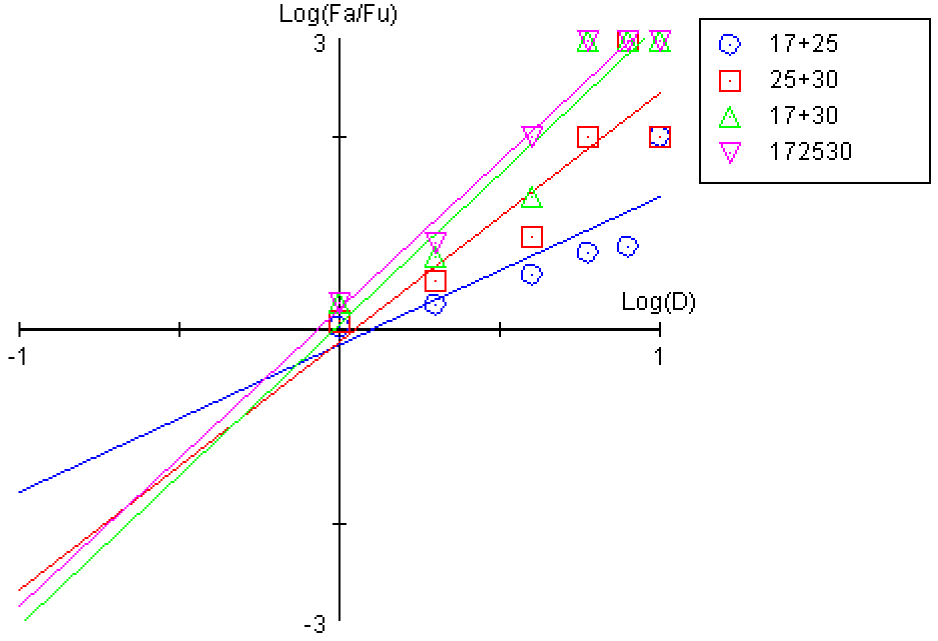

CI Data for Drug Combo: 17+25 (17+25 [1:1])

Fa	CI Value	Total Dose
0.05	62.8050	0.18112
0.1	23.2188	0.29629
0.15	12.6061	0.40185
0.2	7.99573	0.50547
0.25	5.51362	0.61093
0.3	4.00226	0.72092
0.35	3.00534	0.83788

Fa	CI Value	Total Dose
0.4	2.30984	0.96444
0.45	1.80417	1.10373
0.5	1.42478	1.25970
0.55	1.13305	1.43771
0.6	0.90431	1.64534
0.65	0.72214	1.89387
0.7	0.57520	2.20113
0.75	0.45544	2.59740
0.8	0.35695	3.13930
0.85	0.27517	3.94885
0.9	0.20618	5.35563
0.95	0.14499	8.76115
0.97	0.11989	12.4351

CI values for actual experimental points:

Total Dose	Fa	CI Value
10.0	0.99	0.03368
8.0	0.88	0.39745
6.0	0.86	0.37529
4.0	0.79	0.49777
2.0	0.65	0.76261
1.0	0.52	0.97838

CI Data for Drug Combo: 25+30 (25+30 [1:1])

Fa	CI Value	Total Dose
0.05	1.86734	0.35263
0.1	1.42790	0.47150
0.15	1.21390	0.56441
0.2	1.07622	0.64626
0.25	0.97550	0.72273
0.3	0.89603	0.79692
0.35	0.83004	0.87087
0.4	0.77315	0.94627
0.45	0.72263	1.02470
0.5	0.67666	1.10784
0.55	0.63391	1.19774
0.6	0.59335	1.29700
0.65	0.55408	1.40929
0.7	0.51527	1.54008
0.75	0.47598	1.69815
0.8	0.43501	1.89912
0.85	0.39053	2.17452
0.9	0.33901	2.60301
0.95	0.27084	3.48050

Fa	CI Value	Total Dose
0.97	0.23150	4.27969

CI values for actual experimental points:

Total Dose	Fa	CI Value
10.0	0.99	0.25329
8.0	0.999	0.04325
6.0	0.99	0.15197
4.0	0.9	0.52095
2.0	0.76	0.53968
1.0	0.55	0.52926

CI Data for Drug Combo: 17+30 (17+30 [1:1])

Fa	CI Value	Total Dose
0.05	130.018	0.37455
0.1	37.5553	0.47635
0.15	17.5906	0.55280
0.2	10.0369	0.61836
0.25	6.37774	0.67833
0.3	4.33634	0.73546
0.35	3.08825	0.79151
0.4	2.27466	0.84781
0.45	1.71864	0.90556
0.5	1.32462	0.96596
0.55	1.03728	1.03039
0.6	0.82277	1.10057
0.65	0.65938	1.17886
0.7	0.53266	1.26870
0.75	0.43256	1.37555
0.8	0.35176	1.50896
0.85	0.28446	1.68791
0.9	0.22516	1.95882
0.95	0.16558	2.49120
0.97	0.13669	2.95598

CI values for actual experimental points:

Total Dose	Fa	CI Value
10.0	0.999	0.04977
8.0	0.999	0.03982
6.0	0.999	0.02986
4.0	0.96	0.22629
2.0	0.85	0.33706
1.0	0.66	0.52809

CI Data for Drug Combo: 172530 (17+25+30 [1:1:1])

Fa	CI Value	Total Dose
0.05	75.7193	0.32489

Fa	CI Value	Total Dose
0.1	22.1712	0.41511
0.15	10.5225	0.48311
0.2	6.08635	0.54157
0.25	3.92339	0.59515
0.3	2.70836	0.64628
0.35	1.95991	0.69651
0.4	1.46795	0.74705
0.45	1.12856	0.79894
0.5	0.88547	0.85329
0.55	0.70599	0.91133
0.6	0.57006	0.97464
0.65	0.46476	1.04535
0.7	0.38145	1.12660
0.75	0.31404	1.22339
0.8	0.25808	1.34443
0.85	0.20989	1.50711
0.9	0.16586	1.75401
0.95	0.12014	2.24106
0.97	0.09780	2.66794

CI values for actual experimental points:

Total Dose	Fa	CI Value
10.0	0.999	0.03604
8.0	0.999	0.02884
6.0	0.999	0.02163
4.0	0.99	0.06780
2.0	0.89	0.20610
1.0	0.66	0.42104

Combination Index Plot

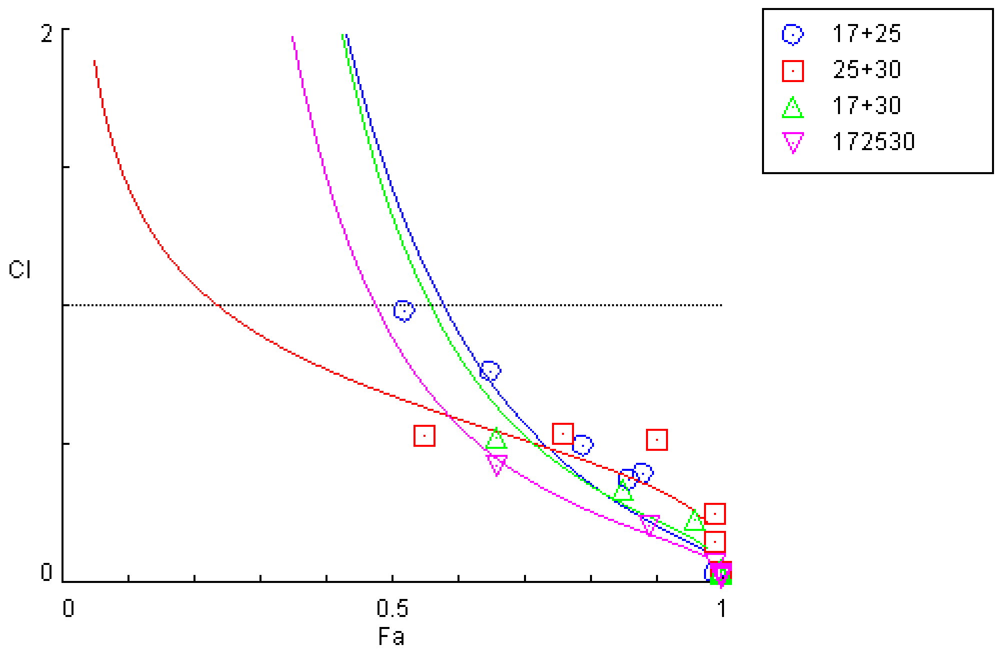

Logarithmic Combination Index Plot

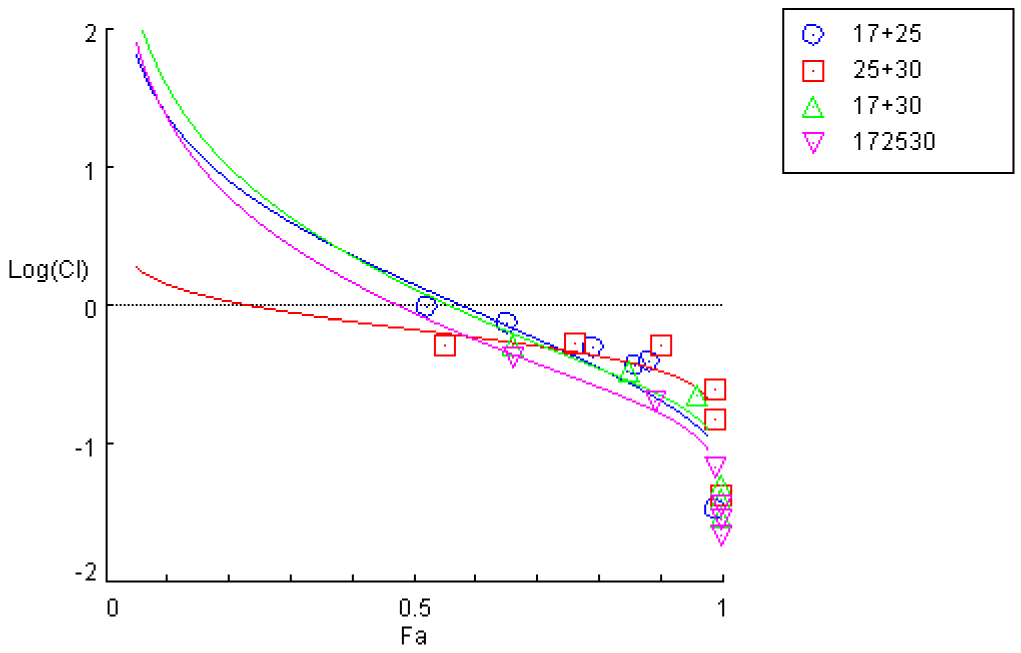

DRI Data for Drug Combo: 17+25 (17+25 [1:1])

Fa	Dose 17	Dose 25	DRI 17	DRI 25
0.05	0.00145	0.20322	0.01604	2.24398
0.1	0.00649	0.39174	0.04378	2.64426
0.15	0.01638	0.58813	0.08154	2.92712
0.2	0.03291	0.79861	0.13022	3.15987
0.25	0.05856	1.02820	0.19170	3.36601
0.3	0.09687	1.28217	0.26874	3.55706

Fa	Dose 17	Dose 25	DRI 17	DRI 25
0.35	0.15301	1.56682	0.36524	3.73996
0.4	0.23469	1.89012	0.48669	3.91961
0.45	0.35370	2.26262	0.64091	4.09997
0.5	0.52866	2.69874	0.83935	4.28474
0.55	0.79018	3.21892	1.09923	4.47785
0.6	1.19086	3.85329	1.44756	4.68390
0.65	1.82656	4.64839	1.92892	4.90888
0.7	2.88522	5.68035	2.62157	5.16130
0.75	4.77285	7.08342	3.67510	5.45425
0.8	8.49200	9.11977	5.41012	5.81006
0.85	17.0599	12.3837	8.64041	6.27205
0.9	43.0900	18.5920	16.0915	6.94297
0.95	192.451	35.8395	43.9328	8.18146
0.97	558.175	57.1705	89.7740	9.19501

DRI values calculated at experimental points

Fa	Dose 17	Dose 25	DRI 17	DRI 25
0.99	5249.53	152.771	1049.91	30.5541
0.88	28.5918	15.5312	7.14794	3.88279
0.86	20.0521	13.2932	6.68404	4.43108
0.79	7.50983	8.64120	3.75491	4.32060
0.65	1.82656	4.64839	1.82656	4.64839
0.52	0.62059	2.89530	1.24117	5.79061

DRI Data for Drug Combo: 25+30 (25+30 [1:1])

Fa	Dose 25	Dose 30	DRI 25	DRI 30
0.05	0.20322	0.17636	1.15259	1.00026
0.1	0.39174	0.28538	1.66167	1.21051
0.15	0.58813	0.38444	2.08405	1.36227
0.2	0.79861	0.48113	2.47152	1.48897
0.25	1.02820	0.57907	2.84531	1.60245
0.3	1.28217	0.68082	3.21783	1.70864
0.35	1.56682	0.78865	3.59828	1.81117
0.4	1.89012	0.90496	3.99488	1.91268
0.45	2.26262	1.03256	4.41618	2.01534
0.5	2.69874	1.17503	4.87206	2.12129
0.55	3.21892	1.33715	5.37500	2.23281
0.6	3.85329	1.52570	5.94185	2.35266
0.65	4.64839	1.75070	6.59676	2.48451
0.7	5.68035	2.02798	7.37671	2.63360
0.75	7.08342	2.38431	8.34250	2.80812
0.8	9.11977	2.86970	9.60422	3.02214
0.85	12.3837	3.59144	11.3899	3.30321
0.9	18.5920	4.83814	14.2850	3.71734

Fa	Dose 25	Dose 30	DRI 25	DRI 30
0.95	35.8395	7.82884	20.5944	4.49868
0.97	57.1705	11.0260	26.7171	5.15272

DRI values calculated at experimental points

Fa	Dose 25	Dose 30	DRI 25	DRI 30
0.99	152.771	22.6696	30.5541	4.53391
0.999	1163.72	100.480	290.930	25.1199
0.99	152.771	22.6696	50.9235	7.55652
0.9	18.5920	4.83814	9.29599	2.41907
0.76	7.42793	2.46881	7.42793	2.46881
0.55	3.21892	1.33715	6.43783	2.67431

DRI Data for Drug Combo: 17+30 (17+30 [1:1])

Fa	Dose 17	Dose 30	DRI 17	DRI 30
0.05	0.00145	0.17636	0.00775	0.94171
0.1	0.00649	0.28538	0.02723	1.19818
0.15	0.01638	0.38444	0.05927	1.39087
0.2	0.03291	0.48113	0.10645	1.55614
0.25	0.05856	0.57907	0.17265	1.70734
0.3	0.09687	0.68082	0.26342	1.85141
0.35	0.15301	0.78865	0.38663	1.99277
0.4	0.23469	0.90496	0.55364	2.13480
0.45	0.35370	1.03256	0.78117	2.28048
0.5	0.52866	1.17503	1.09459	2.43287
0.55	0.79018	1.33715	1.53376	2.59544
0.6	1.19086	1.52570	2.16408	2.77256
0.65	1.82656	1.75070	3.09886	2.97016
0.7	2.88522	2.02798	4.54831	3.19694
0.75	4.77285	2.38431	6.93954	3.46670
0.8	8.49200	2.86970	11.2555	3.80355
0.85	17.0599	3.59144	20.2142	4.25550
0.9	43.0900	4.83814	43.9958	4.93985
0.95	192.451	7.82884	154.505	6.28519
0.97	558.175	11.0260	377.658	7.46014

DRI values calculated at experimental points

Fa	Dose 17	Dose 30	DRI 17	DRI 30
0.999	538064.	100.480	107613.	20.0959
0.999	538064.	100.480	134516.	25.1199
0.999	538064.	100.480	179355.	33.4932
0.96	307.273	9.10011	153.636	4.55006
0.85	17.0599	3.59144	17.0599	3.59144
0.66	1.99585	1.80132	3.99170	3.60265

DRI Data for Drug Combo: 172530 (17+25+30 [1:1:1])

Fa	Dose 17	Dose 25	Dose 30	DRI 17	DRI 25	DRI 30

Fa	Dose 17	Dose 25	Dose 30	DRI 17	DRI 25	DRI 30
0.05	0.00145	0.20322	0.17636	0.01341	1.87648	1.62848
0.1	0.00649	0.39174	0.28538	0.04688	2.83112	2.06243
0.15	0.01638	0.58813	0.38444	0.10173	3.65212	2.38727
0.2	0.03291	0.79861	0.48113	0.18231	4.42389	2.66518
0.25	0.05856	1.02820	0.57907	0.29517	5.18290	2.91895
0.3	0.09687	1.28217	0.68082	0.44966	5.95178	3.16034
0.35	0.15301	1.56682	0.78865	0.65905	6.74855	3.39684
0.4	0.23469	1.89012	0.90496	0.94248	7.59038	3.63413
0.45	0.35370	2.26262	1.03256	1.32812	8.49606	3.87722
0.5	0.52866	2.69874	1.17503	1.85868	9.48825	4.13118
0.55	0.79018	3.21892	1.33715	2.60119	10.5963	4.40177
0.6	1.19086	3.85329	1.52570	3.66556	11.8607	4.69620
0.65	1.82656	4.64839	1.75070	5.24196	13.3402	5.02426
0.7	2.88522	5.68035	2.02798	7.68296	15.1261	5.40024
0.75	4.77285	7.08342	2.38431	11.7040	17.3700	5.84683
0.8	8.49200	9.11977	2.86970	18.9493	20.3502	6.40355
0.85	17.0599	12.3837	3.59144	33.9588	24.6506	7.14901
0.9	43.0900	18.5920	4.83814	73.6995	31.7990	8.27498
0.95	192.451	35.8395	7.82884	257.625	47.9766	10.4801
0.97	558.175	57.1705	11.0260	627.648	64.2862	12.3984

DRI values calculated at experimental points

Fa	Dose 17	Dose 25	Dose 30	DRI 17	DRI 25	DRI 30
0.999	538064.	1163.72	100.480	161419.	349.116	30.1439
0.999	538064.	1163.72	100.480	201774.	436.395	37.6799
0.999	538064.	1163.72	100.480	269032.	581.860	50.2398
0.99	5249.53	152.771	22.6696	3937.15	114.578	17.0022
0.89	34.8140	16.9319	4.51744	52.2210	25.3979	6.77616
0.66	1.99585	4.83264	1.80132	5.98755	14.4979	5.40397

DRI Plot for Combo: 17+25 (17+25 [1:1])

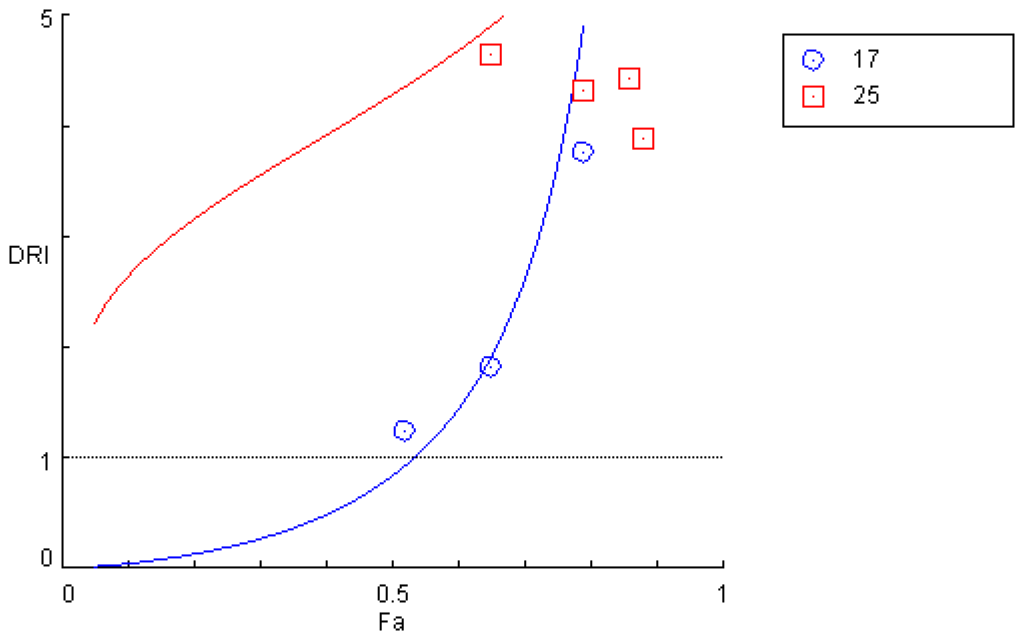

DRI Plot for Combo: 25+30 (25+30 [1:1])

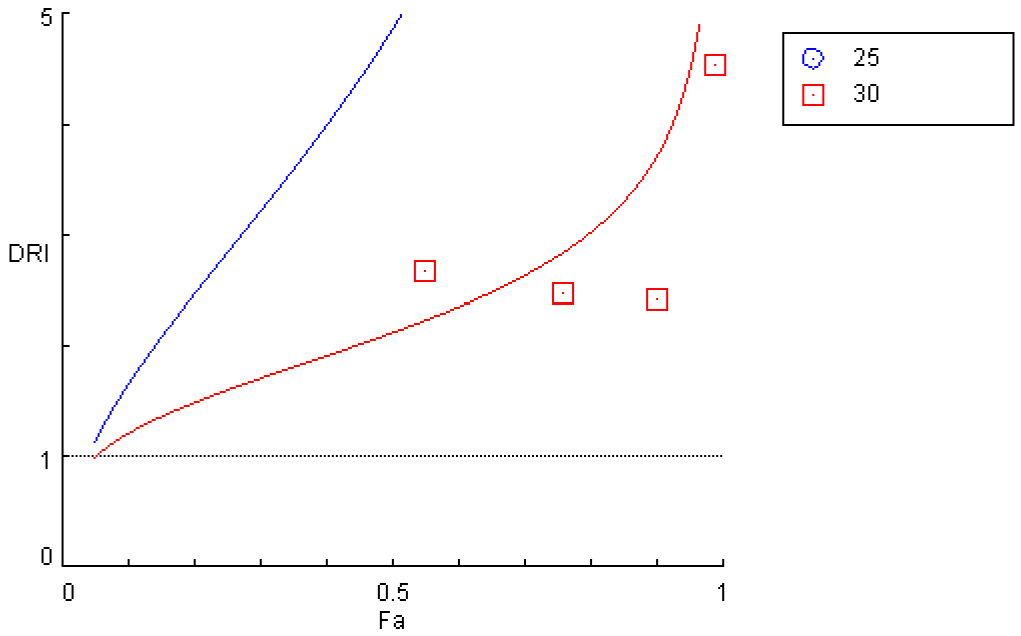

DRI Plot for Combo: 17+30 (17+30 [1:1])

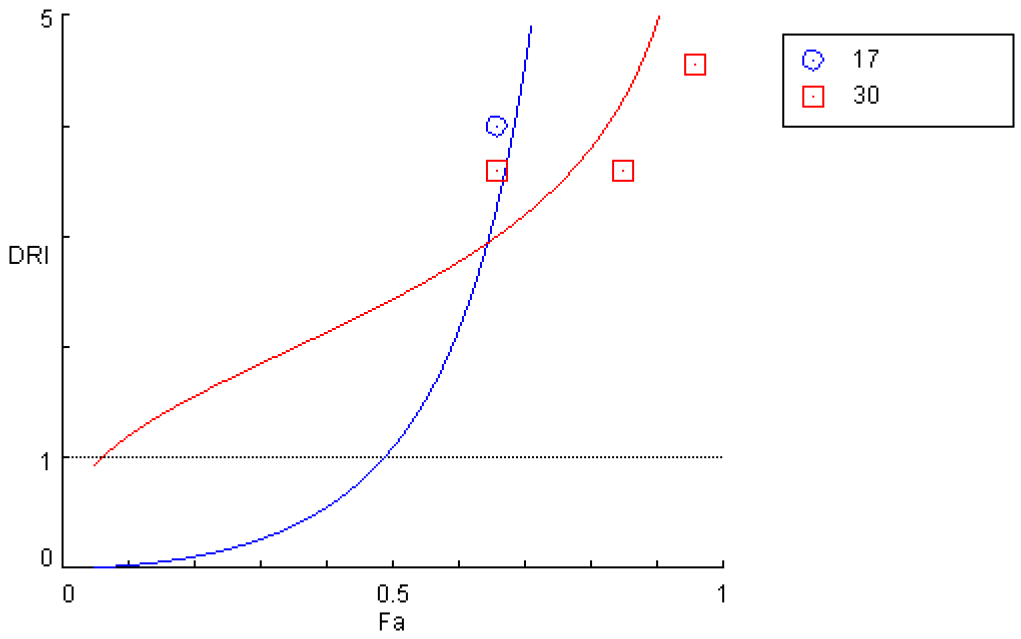

DRI Plot for Combo: 172530 (17+25+30 [1:1:1])

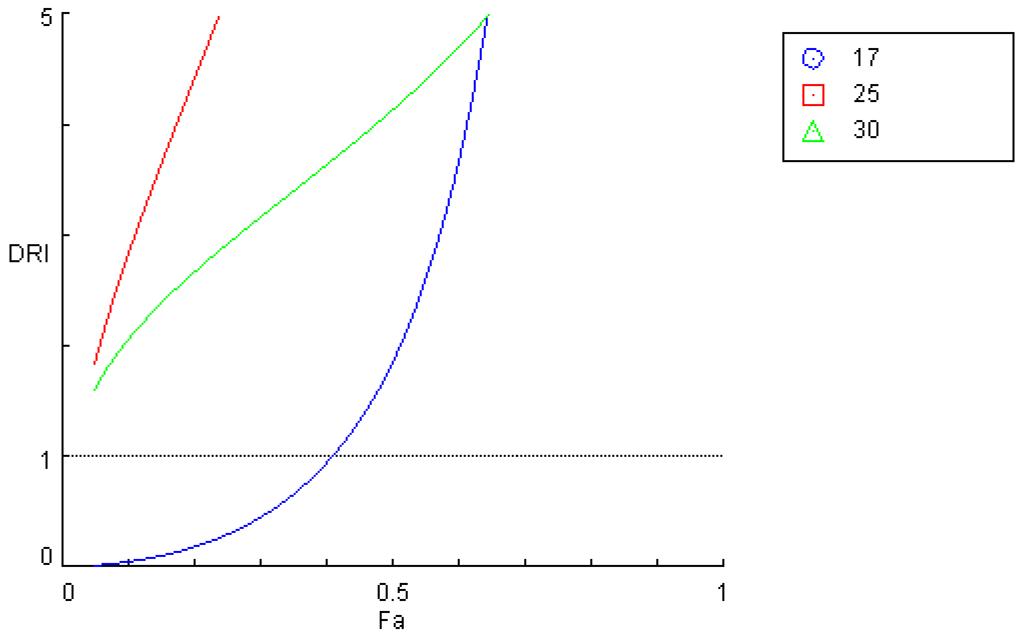

Log(DRI) Plot for Combo: 17+25 (17+25 [1:1])

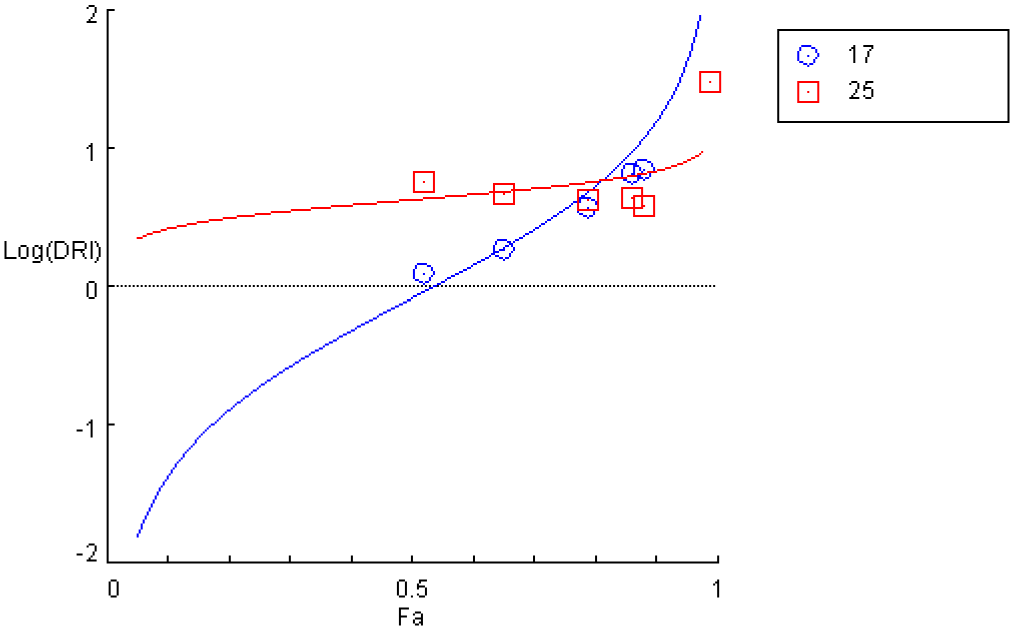

Log(DRI) Plot for Combo: 25+30 (25+30 [1:1])

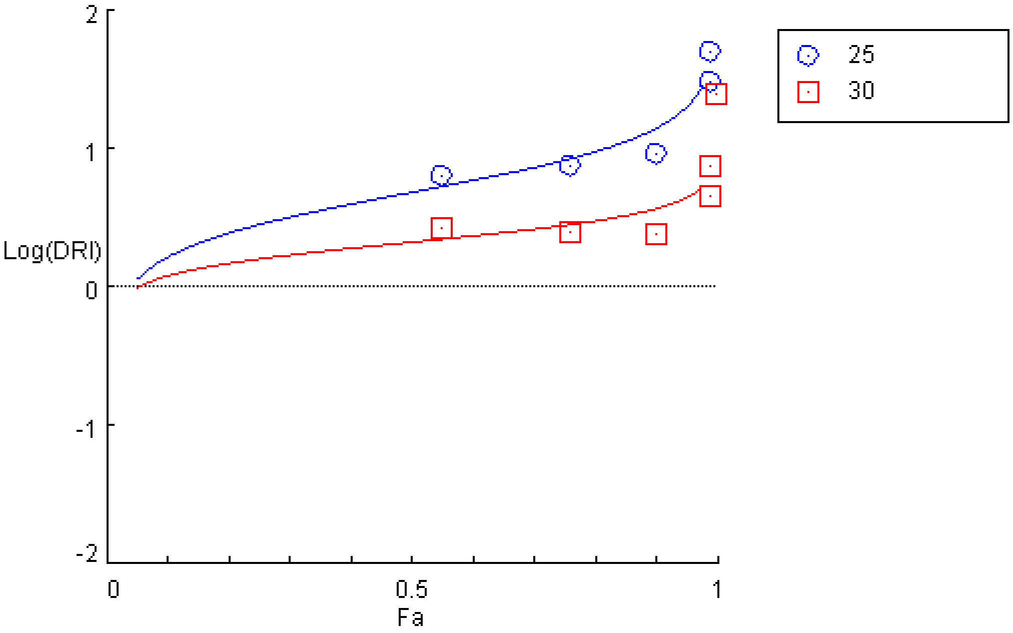

Log(DRI) Plot for Combo: 17+30 (17+30 [1:1])

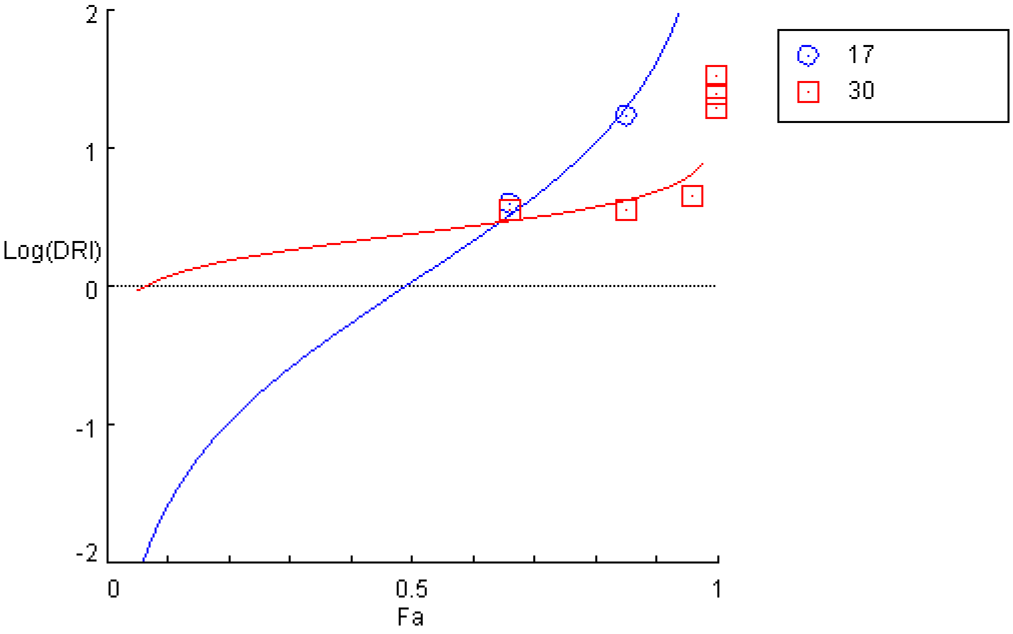

Log(DRI) Plot for Combo: 172530 (17+25+30 [1:1:1])

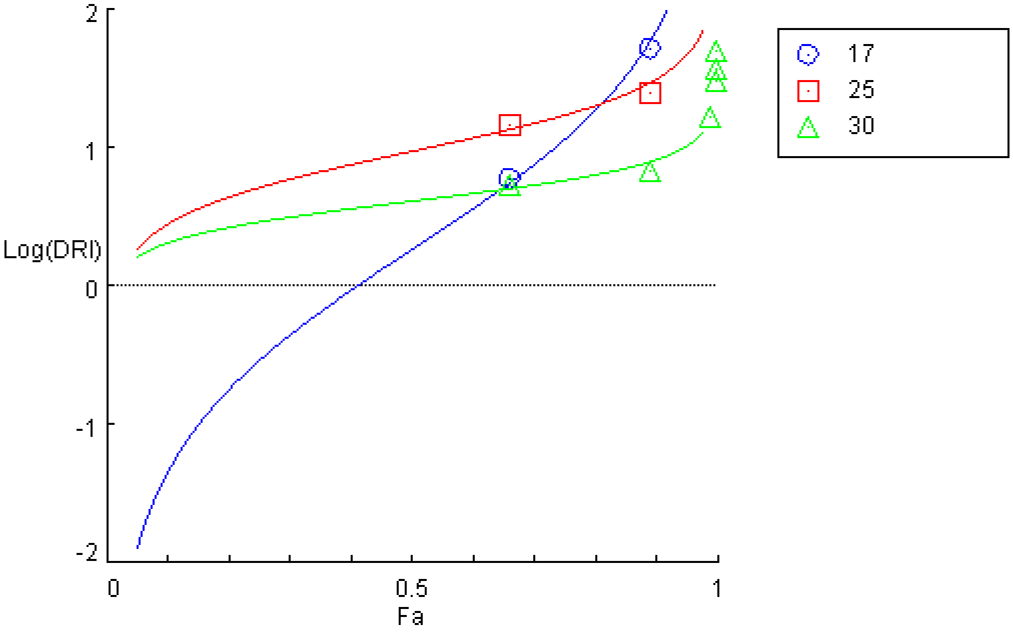

Isobologram for Combo: 17+25 (17+25 [1:1])

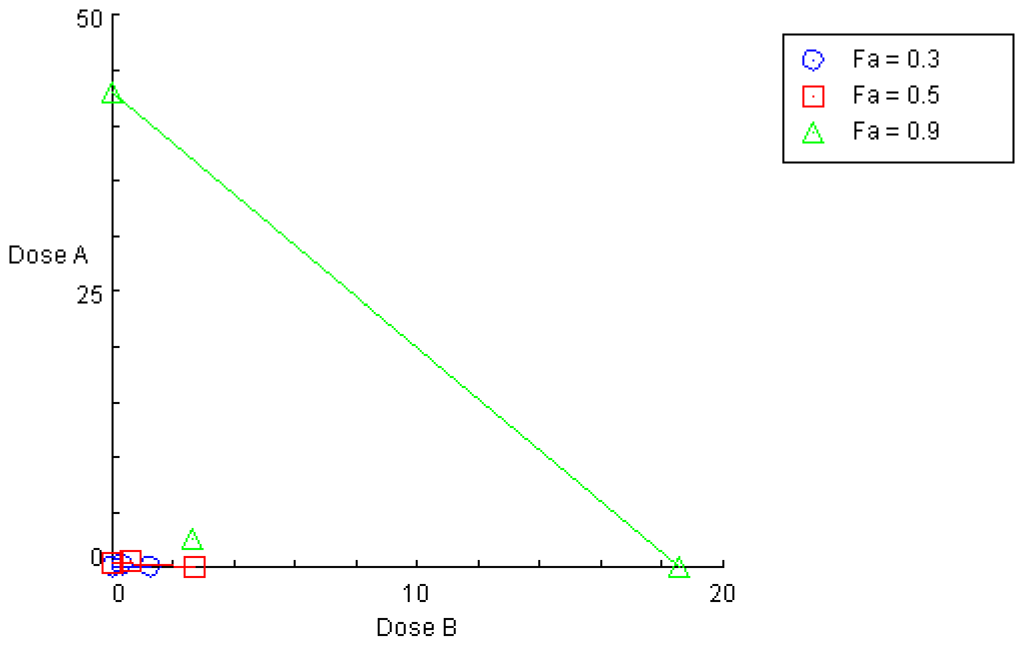

Isobologram for Combo: 25+30 (25+30 [1:1])

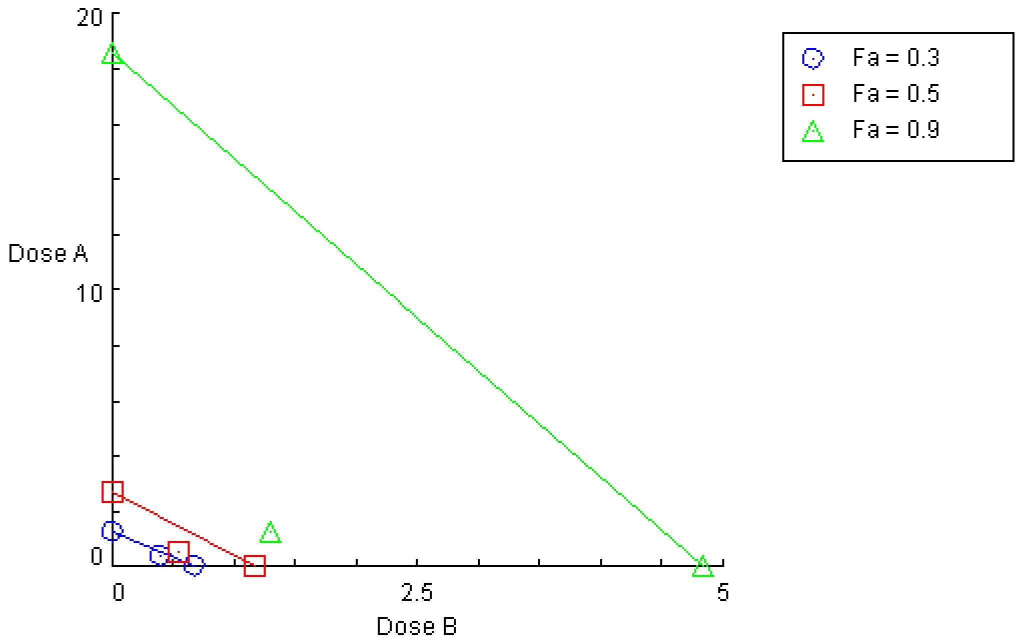

Isobologram for Combo: 17+30 (17+30 [1:1])

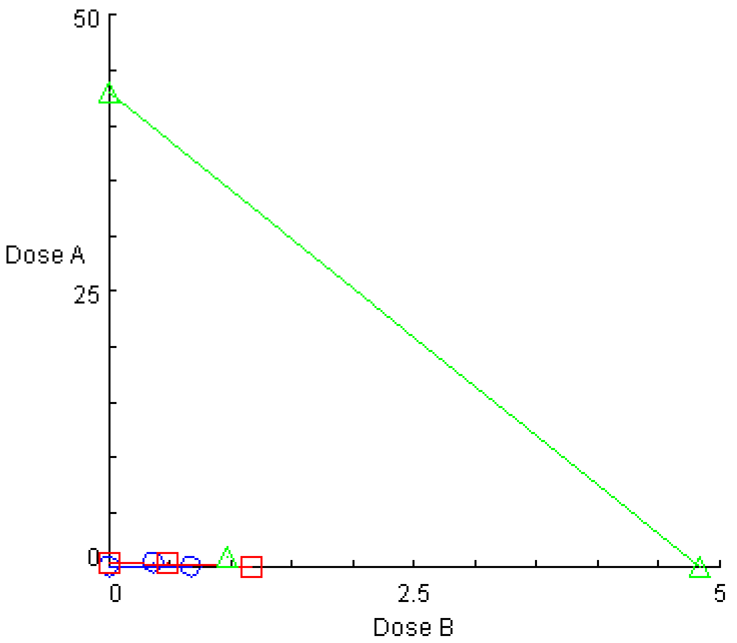

Polygonogram at Fa = 0.9

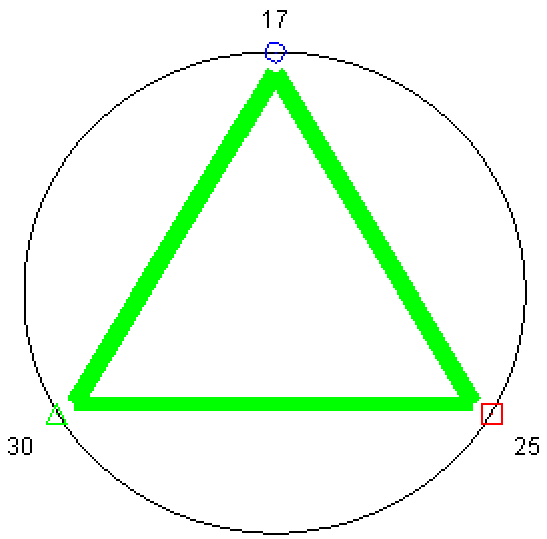

Summary Table

**Experiment Name:**	Four chalcone combinations
**Date:**	1-18-2018
**File Name:**	C:\Users\shant\Desktop\comsyn.cse
**Description**	Synergistic effect of chalcones on M. incognita

**Drug:**	Chalcone (17) [uM]
**Drug:**	Chalcone (25) [uM]
**Drug:**	Chalcone (30) [uM]
**Drug Combo:**	Chalcone (17+25) (17+25 [1:1])
**Drug Combo:**	Chalcone (25+30) (25+30 [1:1])

Drug Combo: Chalcone (17+30) (17+30 [1:1])
Drug Combo: Chalcone 17+25+30 (172530) (17+25+30 [1:1:1])

Drug/Combo	Dm	m	r
17	0.52866	0.49929	0.91179
25	2.69874	1.13849	0.75109
30	1.17503	1.55255	0.96473
17+25	1.25970	1.51818	0.84971
25+30	1.10784	2.57209	0.89924
17+30	0.96596	3.10792	0.95207
172530	0.85329	3.04931	0.97873

CI values at:

Combo	ED50	ED75	ED90	ED95
17+25	1.42478	0.45544	0.20618	0.14499
25+30	0.67666	0.47598	0.33901	0.27084
17+30	1.32462	0.43256	0.22516	0.16558
172530	0.88547	0.31404	0.16586	0.12014

Data for Fa = 0.3

Drug/Combo	CI value	Dose 17	Dose 25	Dose 30
17		0.09687		
25			1.28217	
30				0.68082
17+25	4.00226	0.36046	0.36046	
25+30	0.89603		0.39846	0.39846
17+30	4.33634	0.36773		0.36773
172530	2.70836	0.21543	0.21543	0.21543

Data for Fa = 0.5

Drug/Combo	CI value	Dose 17	Dose 25	Dose 30
17		0.52866		
25			2.69874	
30				1.17503
17+25	1.42478	0.62985	0.62985	
25+30	0.67666		0.55392	0.55392
17+30	1.32462	0.48298		0.48298
172530	0.88547	0.28443	0.28443	0.28443

Data for Fa = 0.75

Drug/Combo	CI value	Dose 17	Dose 25	Dose 30
17		4.77285		
25			7.08342	
30				2.38431
17+25	0.45544	1.29870	1.29870	

Drug/Combo	CI value	Dose 17	Dose 25	Dose 30
25+30	0.47598		0.84908	0.84908
17+30	0.43256	0.68778		0.68778
172530	0.31404	0.40780	0.40780	0.40780

Data for Fa = 0.9

Drug/Combo	CI value	Dose 17	Dose 25	Dose 30
17		43.0900		
25			18.5920	
30				4.83814
17+25	0.20618	2.67782	2.67782	
25+30	0.33901		1.30151	1.30151
17+30	0.22516	0.97941		0.97941
172530	0.16586	0.58467	0.58467	0.58467

Data for Fa = 0.97

Drug/Combo	CI value	Dose 17	Dose 25	Dose 30
17		558.175		
25			57.1705	
30				11.0260
17+25	0.11989	6.21755	6.21755	
25+30	0.23150		2.13984	2.13984
17+30	0.13669	1.47799		1.47799
172530	0.09780	0.88931	0.88931	0.88931
